# Elevated monocyte phosphorylated p38 in nearby employees after a chemical explosion

**DOI:** 10.1038/srep29060

**Published:** 2016-07-06

**Authors:** André Sulen, Stein H. L. Lygre, Sigrun M. Hjelle, Bjørg E. Hollund, Bjørn T. Gjertsen

**Affiliations:** 1 Centre for Cancer Biomarkers CCBIO, Department of Clinical Science, University of Bergen, Bergen, N-5021 Norway; 2 Department of Occupational Medicine, Haukeland University Hospital, Bergen, N-5021 Norway; 3 Department of Internal Medicine, Haematology Section, Haukeland University Hospital, Bergen, N-5021 Norway

## Abstract

Personalised health surveillance is infrequent or absent in occupational and environmental medicine. The shortage of functional tests in relevant cells and tissues greatly limits our understanding of environmental exposures and associated disease risk. We evaluated single cell signalling in peripheral blood mononuclear cells from 301 individuals in a cross sectional health survey 18 months after a chemical explosion of sulphorous coker gasoline. The accident created a malodourous environment leading to long-term health complaints. Multiple regression analysis revealed T-cell specific elevated phosphorylation of the stress kinase p-p38 (T180/Y182) among tobacco smokers and monocyte-specific elevated phosphorylation in employees at the explosion site. Other studies of the accident reported reduced tear film stability, and more airway obstruction and subjective health complaints among the employees at the accident site. Elevated monocyte p-p38 in the employee group was independent of such health effects, and could therefore be dependent on the sulphuric malodorous environment. The present study proposes signalling status in leukocytes as a scalable biomarker providing information about environmental exposures.

Improved methods and new biomarkers for assessing impact of environmental exposures on biological processes have been called for^1^,^2^,^3^. Environmental and occupational pollutants, such as hydrogen sulfide (H_2_S), hydrocarbons and particles, are known to modulate intracellular signalling^4^,^5^,^6^,^7^,^8^,^9^. However, studies of individual exposure based on single cell molecular signalling responses in the test person’s leukocytes have not been performed. *In vitro* studies show that aromatic hydrocarbons mediate increased cellular oxidative stress^4^,^5^. This contributes to inflammatory signalling via the stress kinase p38 MAP Kinase (MAPK) which influences prostaglandin synthesis and production of various cytokines^10^,^11^,^12^,^13^,^14^. Tobacco smoke, containing aromatic hydrocarbons and tar particles, is the major source of environmental exposure to benzene and is known to activate p38 in an oxidative stress dependent way^15^,^16^,^17^,^18^. Phosphorylation of p38 (T180/Y182) indicates p38 activation and is therefore a potential biomarker of exposure to both particles and a wide range of chemicals^4^,^5^,^6^,^7^. Single cell analysis of phospho-signalling enzymes has been used to delineate heterogeneous material and detect unique signalling profiles in cellular subsets of clinical patients^19^,^20^. This methodology has however not been employed to evaluate variation in immune cell signalling in a cross sectional population study related to environmental pollution.

The natural variation of p-p38 in leukocytes in a healthy population is not known, but endogenous regulators of p38-activity change by age, body mass index (BMI) and exposure to cigarette smoke^21^,^22^,^23^,^24^. Levels of the p38-modulating cytokines interleukin-1 (IL-1), tumor necrosis factor alpha (TNF-α) and the IL-1 receptor antagonist (IL-1ra) are known to vary between different age groups^21^. Also, the adipokine leptin is found to correlate with BMI and percentage body fat in humans^22^,^23^. Leptin-mediated monocyte activation is p38 dependent and leads to increased production of TNF-α^25^. In addition, smokers have higher levels of cytokines relevant to p38 signalling compared to non-smokers^24^. Therefore, in addition to environmental stress, age and BMI may also influence the level of p-p38 in leukocytes.

The present study is based on a cohort of healthy individuals who took part in a cross sectional survey in the aftermath of a chemical explosion^26^,^27^,^28^,^29^,^30^. In May 2007, a tank containing sulphorous coker gasoline exploded in the Gulen municipality of western Norway. In addition to the combustion of chemicals during the following fire, chemical material was spread to the immediate area during the initial explosion. The fire was extinguished within 24 hours, however, an intense malodorous smell of sulphur remained in the area for two years^29^,^30^. At the start-up of a health survey initiated 18 months after the explosion, exposure assessment indicated that H_2_S and mercaptans remained present^28^. Other studies related to the survey found that the group of exposed workers had more airway obstruction, subjective health complaints and reduced tear film stability compared to people living >20 kilometres away from the polluted area^27^,^28^,^30^.

The aim of the present study was to assess the population based variation of phosphorylated p38 stress kinase (pT180/pY182) in blood cells collected during this population survey in Gulen. Monocyte p-p38 was found to be higher in employees at the malodourous explosion site compared to people living >20 km away. The results indicate a role for single cell signalling profiling in future personalised exposure monitoring.

## Results

### Single cell analysis of phosphorylated p38 in study group

**Figure 1:**
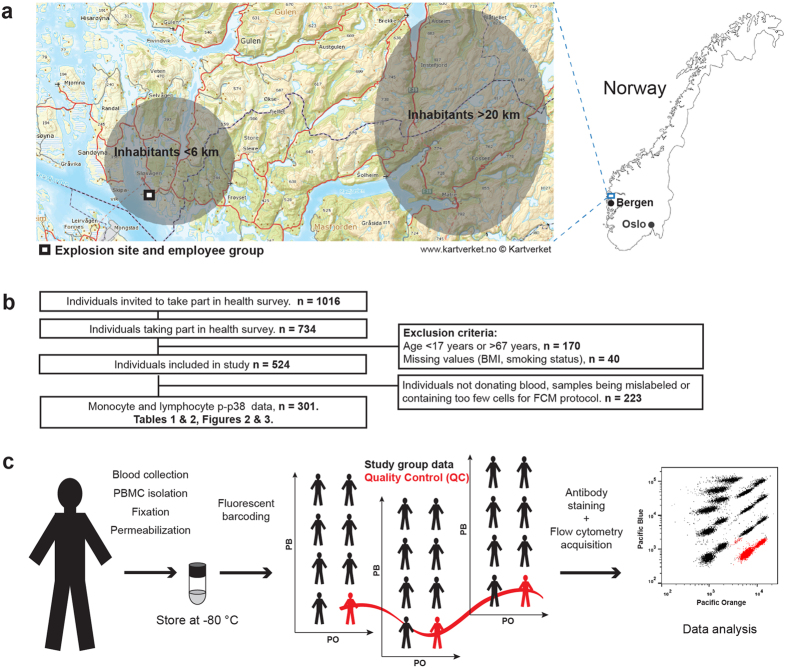
Health survey participants included in the current study group. (**a**) Geographic distribution of individuals participating in the health survey in 2008–2009 and in the current study group; (i) gender and age matched inhabitants living >20 km from the site, (ii) nearby inhabitants living <6 km from the site and (iii) individuals employed at the site. (**b**) Flow chart describing number of participants from the health survey included in the current study group. Children (age <17) or participants above the retirement age (age >67) were excluded from the study group. (**c**) Overview of phospho flow workflow (details provided in materials and methods). The detailed map in (a) was downloaded from www.norgeskart.no and is licensed by The Norwegian Mapping Authority (^©^Kartverket, www.kartverket.no) under Creative Commons Attribution 4.0 International (CC BY 4.0, http://creativecommons.org/licenses/by/4.0/). The image of Norway is under Public domain (CC0, http://creativecommons.org/publicdomain/zero/1.0/deed.en) and is downloaded from http://publicdomainvectors.org. Both maps have been modified by adding names of cities or indicating locations of groups of individuals taking part in the study.

**Figure 2:**
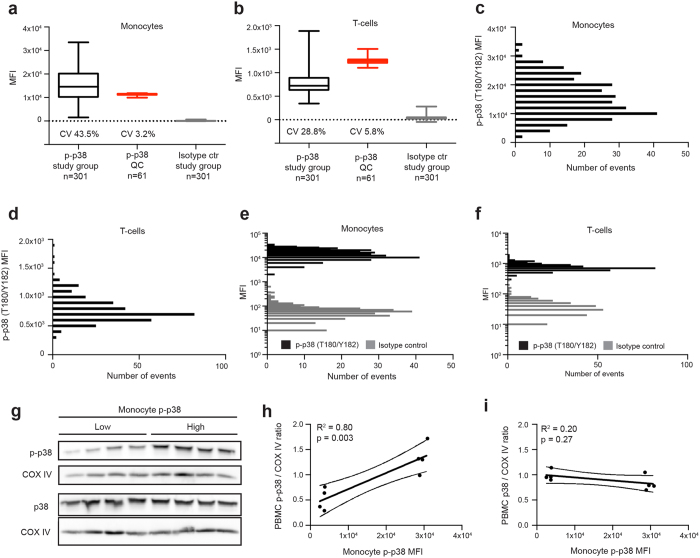
Descriptive data of p-p38 single cell analysis in the study group. (**a**,**b**) Box plots displaying MFIs of study group p-p38, quality control (QC) p-p38 and study group isotype control for monocytes (**a**) and T-cells (**b**). Boxes extend from 25th to 75th percentiles; whiskers display minimum to maximum values and middle line represents the median. (**c**) Histogram displaying distribution of monocyte p-p38 MFI (binning width 2,000). (**d**) Histogram displaying distribution of T-cell p-p38 MFI (binning width 100). (**e**) Histogram comparing p-p38 and isotype control MFIs for monocytes (binning width is 2,000 and 10 for p-p38 and isotype, respectively). (**f**) Histogram comparing p-p38 and isotype control MFIs for T-cells (binning width is 100 and 10 for p-p38 and isotype, respectively). (**g**) Cropped western blots of PBMC lysates, from eight individuals with high or low monocyte p-p38, probed with antibodies detecting p-p38, total p38 and COX IV. Uncropped images are available in Supplementary Fig. S2. (**h**,**i**) Dot plots comparing monocyte p-p38 MFI to p-p38/COX IV ratios (**h**) and p38/COX IV ratios (**i**) determined by quantitation of western blots. Solid lines represent slopes of linear regressions (curves represent 95% CI).

Fixed and methanol-permeabilised peripheral blood mononuclear cells (PBMCs) from the study group (Fig. 1) were barcoded, stained with antibodies and analysed by flow cytometry (overview of workflow in Fig. 1c). The selected antibody panel enabled simultaneous assessment of immune phenotype and signal transduction in single cells. Fluorescent barcoding facilitated high throughput data collection with reduced antibody consumption. A reoccurring quality control sample (QC) was included to determine data quality in each barcoded group (Fig. 1c). T-cells and monocytes were identified as CD5+ and CD163+, respectively. CD163 expression was determined to comprise predominantly classical monocytes (Supplementary Fig. S1). For the study group p-p38 median fluorescent intensities (MFIs) averaged at 15,246 ± 6,624 and 772 ± 222 for monocytes and T-cells, respectively (mean ± SD) (Fig. 2a,b). By measuring p-p38 repeatedly for the QC sample in the barcoded material, it was possible to determine its coefficient of variation (CV) to describe the intrinsic variation in the assay. The QC CV was 3.2% and 5.8% for monocytes and T-cells, respectively, demonstrating low variation introduced by fluorescent barcoding, antibody staining and acquisition (red boxes in Fig. 2a,b). In general, monocytes displayed a higher p-p38 signal and a wider distribution compared to the T-cells (Fig. 2c,d). Indicative of a high signal-to-noise ratio in the flow cytometry assay, cells stained with isotype control showed a markedly lower MFI compared to the same samples stained with the p-p38 antibody (Fig. 2e,f). Further, protein lysates of PBMCs from individuals with high or low monocyte p-p38 MFIs were analysed on western blots and evaluated for p-p38 and total p38 signal (Fig. 2g). Comparing quantitated bands from the p-p38 western blot with monocyte p-p38 MFIs demonstrates specificity for the flow cytometry data (Fig. 2h). Comparing quantitated bands from the total p38 western blot with monocyte p-p38 MFIs indicates low variance in expression of the protein p38 (Fig. 2i).

### Elevated monocyte p-p38 in individuals employed at the explosion site

**Figure 3:**
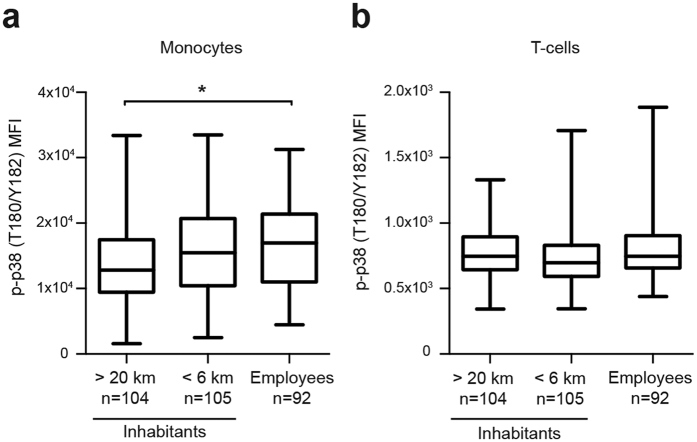
Selective elevated monocyte p-p38 in individuals employed at the explosion site. Box plots displaying p-p38 MFIs in each geographical subgroup for monocytes (**a**) and T-cells (**b**). Boxes extend from 25th to 75th percentiles; whiskers display minimum to maximum values and middle line represents the median. ANOVA with Dunnett’s multiple comparison post hoc tests was used to assess statistical differences between groups (*p < 0.05).

Initial assessment of p-p38 variation indicated no significant association between monocyte p-p38 and living within 6 km from the explosion site compared to individuals living >20 km away (Dunnett´s test, p = 0.13) (Fig. 3a). Employees at the explosion site had elevated monocyte p-p38 compared to individuals living >20 km away (Dunnett´s test, p = 0.01) (Fig. 3a). No significant association between T-cell p-p38 and working or living close to the explosion site was found compared to individuals living >20 km away (Fig. 3b). To further assess the variation in p-p38, a multiple linear regression analysis with monocyte p-p38 as the dependent variable and gender, age, smoking habits, geographic groups and BMI as independent variables was carried out (Table 1). This regression analysis confirmed significant association between monocyte p-p38 and being employed at the explosion site compared to living >20 km away (β = 2,711, p = 0.006). Monocyte p-p38 for those living close to the explosion site was not significantly different from those living >20 km away (β = 1,605, p = 0.08). No significant association was found between monocyte p-p38 and age, gender, smoking habits or BMI.

### Elevated T-cell p-p38 in tobacco smokers

Multiple linear regression analysis with T-cell p-p38 as a dependent variable confirms no association between T-cell p-p38 and being employed or living close to the accident site compared to living >20 km away (Table 2). The regression analysis shows significant association between T-cell p-p38 and tobacco smokers compared to non-smokers (β = 74, p = 0.01). No significant association was found between T-cell p-p38 and age, gender or BMI. However, a tendency of elevated p-p38 was observed in both monocytes and T-cells for those with a high BMI (Tables 1 and 2). These tendencies were diminished if CRP was included in the models (Supplementary Tables S2 and S3).

Performing the same analyses stratified by gender implied a higher level of T-cell p-p38 among male smokers compared to male non-smokers, and a higher level of monocyte p-p38 among male workers and female inhabitants as compared to their respective reference groups (Tables 3 and 4). Testing for interaction between gender and smoking habits on T-cell p-p38 (Table 3), and between gender and geographic group on the level of monocyte p-p38 (Table 4) did not reach levels of statistical significance in the unstratified models.

### C-reactive protein, healthy worker effect or other reported health effects are not likely confounders

Inflammatory diseases and mild infections are known to increase levels of cytokines relevant to p38 signalling^31^. Serum C-reactive protein (hs-CRP) for the group of individuals in the regression analyses averaged at 2.37 ± 2.81 mg/L ((mean ± SD), range 0–21 mg/L). CRP was not significantly associated with p-p38 when included in the regression analyses or influenced the significance of the other predictors (Supplementary Tables S2 and S3). Infections were therefore not considered to be an important confounder for elevated p-p38 in employees or smokers. The employee group did not show substantially lower monocyte p-p38 variance compared to the reference group, and individuals older than the current retirement age in Norway (age 67) were excluded from the study group. We therefore do not consider a healthy worker effect as an important confounder for elevated monocyte p-p38 in the employee group in this study.

Reduced tear film stability, and more airway obstruction and subjective health complaints have been reported for this employee group in other studies^27^,^28^,^30^. Tear film stability (NIBUT), airway obstruction (FEV1/FVC ratio < 0.7) or subjective stressful life events (Impact of Event Scale-Revised; (IES-R)) did not significantly associate with monocyte p-p38 when included in the regression model (Supplementary Tables S4–S6).

## Discussion

Analysis of the basal p38 (pT180/pY182) phosphorylation level indicated elevated monocyte p-p38 in individuals working close to a malodourous environment, determined in a single cell assay suitable for population studies. We also detected significantly elevated p-p38 (pT180/pY182) in T-cells in male smokers compared to male non-smokers; however, no such association was observed for females (Table 3). Elevated leukocyte p-p38 in smokers compared to earlier smokers and non-smokers has previously been reported^32^. This is supported by *in vitro* experiments where exposure of epithelial cells to cigarette smoke extract (CSE) induces ROS-mediated phosphorylation of p-p38^17^. Activation of p38 by CSE influence the anti-oxidative response by increasing the expression of Nrf2 (Nuclear factor (erythroid-derived 2)-like 2)^17^. Tobacco smokers show increased expression of Nrf2-regulated genes in PBMCs, however, this anti-oxidative response is attenuated in heavy smokers compared to moderate smokers^33^. In addition, smoking duration can influence the biological response^34^, but the present study is limited by a lack of information on smoking intensity and duration. This could explain the different results for genders. Second-hand smoke is also not accounted for in the present study which could also influence the results.

Individuals employed at the malodorous explosion site showed significantly elevated p-p38 in monocytes but no modulation of p-p38 in T-cells when compared to individuals living >20 km away (Tables 1 and 2). Individuals living <6 km from the explosion site did show a non-significantly tendency of higher monocyte p-p38 compared to the individuals living >20 km away. After stratification by gender, male employees had a significantly higher monocyte p-p38 compared to their reference group; this was not significant for women (Table 4). The low number of females in this group, making it harder to acquire statistical significance, could explain this. Males may also be more likely to have worked outdoors compared to women, thereby being exposed to higher levels of pollution and for a longer time. This has been suggested in a study reporting reduced tear film stability (NIBUT) among men but not women in the same cohort of employees in the survey^30^. Moen *et al*. suggest that the sulphuric remains from the accident may have lead to irritation of the eyes, but that other types of pollution not related to the accident could also be involved (i.e. dust)^30^. When including tear film stability in the regression analysis in the present study, no significant association between NIBUT and monocyte p-p38 was found (Supplementary Table S4).

Other studies reported more airway obstruction and subjective health complaints among the employees 18 months after the accident^27^,^28^. Both obstructive lung disease and posttraumatic stress disorder (PTSD) is associated with a low-grade systemic inflammation, with increased production of TNF-α or IL-1β in blood cells^35^,^36^,^37^. However, including airway obstruction (FEV_1_/FVC ratio < 0.7) or subjective stressful life events (IES-R) in the regression models did not result in significant associations with monocyte p-p38 (Supplementary Tables S5 and S6). The elevation of monocyte p-p38 in the employee group is therefore likely independent of the health effects reported by others^27^,^28^,^30^. This could indicate elevated monocyte p-p38 to be dependent on the malodorous environment either through uptake of chemicals in the blood or through olfactory perception. Such *in vivo* modulation of stress signalling in peripheral blood leukocytes in individuals exposed to a malodorous environment has not been described previously. Interestingly, recent reports propose a role of olfactory perception and central nervous influence in blood progenitor maintenance that may be relevant for understanding our observations of variation in leukocyte signalling^38^,^39^.

The coker gasoline involved in the explosion accident was reported to contain large amounts of sulphur^27^. Exposure assessment performed at the start of the health survey 18 months after the explosion indicated presence of H_2_S, various mercaptans and volatile organic compounds (VOCs), this co-occurred with the persistent smell in the area during the survey^28^,^29^,^30^. The concentrations of these sulphurous chemicals were likely at sub-toxic levels^28^. Exposure to low levels of H_2_S is common in the paper producing industry, in the processing of crude oil, in sewers and in proximity of geothermal fields^40^,^41^,^42^,^43^,^44^. The malodourous gas H_2_S, which is also an endogenous gasotransmitter, is reported to attenuate oxidative stress and p-p38 in the lungs of rats exposed to passive smoking^45^, and H_2_S alone also attenuates p-p38 in granulocytes^46^. The effect on p-p38 in lymphocytes and monocytes is not reported in the literature but H_2_S does induce phosphorylation of p38 in endothelial cells^47^. Smokers did not show elevated monocyte p-p38 in the present study, and an effect from particles or volatile organic compounds on elevated monocyte p-p38 in the employee group is therefore considered unlikely but cannot be ruled out.

We propose elevated phosphorylation of p38 in monocytes as a possible marker during exposure to a sulphuric malodourous environment and indicate that elevated p-p38 in T-cells could be a marker of smoking. There is a need for new biomarkers in health studies of environmental and occupational exposure^1^,^2^, and p-p38 in monocytes could be used for assessment of biological effects of exposure to a malodorous environment. Future studies should address the ability of single cell signalling profiling to provide risk assessment both at group and at the individual level for long-term health effects.

## Methods

### Study design

The cross sectional study is part of the health survey that was carried out 18 months after the accident described previously^26^,^27^,^28^,^29^,^30^. The aim of the study was to assess the population-based variation in phosphorylated p38 in PBMCs as determined by phospho flow cytometry. During the medical examination of the presumptive healthy individuals taking part in the health survey, blood was drawn and weights and heights were recorded. A questionnaire was answered, informing about smoking habits, current work and subjective stressful life events (IES-R)^48^. Airway function was measured by spirometry, this has been described in other studies^26^,^27^. The measurement of tear film stability (NIBUT) has also been described in another study^30^.

Of the 1,016 individuals invited to take part in the survey, 734 met for the examination (Fig. 1b). 231 individuals lived more than 20 km from the accident site, 249 individuals lived within 6 km from the accident site and the remaining 254 were employees in the accident area. Of these, we included 524 adult participants aged 17–67 years in the present study. Due to mislabelled samples, few cells in the processed samples or some participants not donating blood, 301 participants were included in the final regression analyses (Tables 1 and 2). The local research ethics committee at Haukeland University Hospital (Regionale komiteer for medisinsk og helsefaglig forskningsetikk, REK) approved the study. Recruitment, sample collection, data collection and data analysis were carried out in accordance with the approved ethical guidelines. All individuals included in the study provided written informed consent.

### Sample collection

For the population study a blood sample was taken by venepuncture and collection in Cell Preparation Tubes with sodium citrate (BD Vacutainer ^®^ CPT™). Mononuclear cells were harvested after centrifugation (1,500 rcf, 20 min), this was followed by washing of the cells in 0.9% NaCl. Cells for flow cytometry were fixed in 4% paraformaldehyde (PFA) for 5 minutes at room temperature (RT) prior to permeabilisation in ice cold methanol. For western blot the cells were suspended in 7% ice cold trichloroacetic acid (TCA) for protein precipitation. Samples were stored at −80 °C until transportation on dry ice to the central research lab. Health care personnel involved in the consultations also carried out the initial sample collection. The major findings of the study are cellular subset-specific for monocytes or T-cells in the same sample from the individuals, reducing the probability that sample preparation has biased the results.

### Single cell phospho-p38 analysis

To achieve high throughput and to reduce antibody consumption, the samples from the population study were fluorescently barcoded as described by Krutzik & Nolan^49^. All samples were analysed by grouping seven individual samples and one quality control sample (QC) using four different concentrations of the amine-reactive fluorescent dye Pacific Blue and two different concentrations of Pacific Orange (both from Molecular Probe, Eugene, OR, USA). Intracellular staining was performed based on the work of Skavland *et al*.^50^. In summary, the fixed/permeabilised and barcoded samples were washed with 0.5% BSA in PBS and re-suspended in 0.5% BSA/PBS with 200 μg/ml human IgG (Octagam, Octapharma AG) for blocking. This was followed by antibody staining with monocyte surface marker CD163 PE (Clone MAC2-158, Trillium Diagnostics), T-cell marker CD5 PerCP-Cy5.5 (Clone L17F12, BD) and Anti-p38 MAPK Alexa Fluor^®^ 647 (pT180/pY182, Clone 36/p38, BD). An additional panel using the same surface markers and Mouse IgG1 κ Isotype control Alexa Fluor^®^ 647 (Clone MOPC-21, BD) was used to determine background. The canonical surface markers CD3 (T-cells) and CD14 (monocytes) were damaged by the methanol permeabilisation during sample collection, and were replaced with CD5 and CD163, respectively. CD5 is known to co-express with CD3 51 and was considered an adequate substitute. It should be noted that B1-a cells (subset of b-cells) are reported to express CD5, however, these cells are rare in the peripheral blood of adults^52^. CD163 was after validation considered to detect predominantly classical monocytes (Supplementary Fig. S1), which is in accordance with the work of Tippett and colleagues^53^. It should be noted that PBMCs normally contain 1% dendritic cells^54^, of which 10% are known to express CD163 55, however, this is a minute population compared to the classical monocytes. Following antibody incubation and washing with PBS, the samples were re-suspended in PBS and analysed by flow cytometry. The data was collected on a FACS Fortessa flow cytometer (BD). The PMT setting for the Alexa Fluor 647^®^ channel was set high to increase separation between signal and background. Sufficient events were recorded to ensure a low theoretical standard error of median (σM_D_) for the median fluorescent intensity (MFI) (500 cells, σM_D_ < 0.06). However, for 75% of the samples more cells were recorded (1,500 cells, σM_D_ of 0.032). Gating and export of MFIs were carried out using FlowJo (TreeStar Inc). Raw value MFIs of p-p38 Alexa Fluor 647^®^ were used in the statistical analyses.

### Immunoblot

Precipitated protein samples from the population study were processed and analysed by SDS-PAGE and western blotting as described elsewhere^56^. Membranes were probed with Phospho-p38 MAPK antibody (Thr180/Tyr182, clone: 12F8) and COX IV antibody (loading control), both from Cell Signaling. Peroxidase-conjugated Donkey Anti-Rabbit IgG (H +L) antibody (Jackson ImmunoResearch) was used as secondary antibody and visualized by the use of Supersignal West Pico (Pierce Technology, Inc., IL, USA). Chemiluminescence was detected using an Imagequant LAS4000 imager and the protein bands were quantitated with the use of Imagequant TL Software (both from GE Healthcare Life Sciences).

### C-reactive protein (CRP)

Serum hs-CRP was determined with an immuno assay (Tina-quant^®^/Modular P system (Roche)).

### Statistics

MFIs of p-p38 were used in the statistical analyses without any data pre-processing. Analysis of variance (ANOVA) with Dunnett’s multiple comparison post hoc tests was used for initial assessment of variation in geographic groups in the population study. In addition, multiple linear regressions were carried out in R (www.R-project.org) to examine effects on p-p38 by the categorical predictors gender, age (17–39 years, 40–49 years and 50–67 years), body mass index (low (<25), medium (25–29.9) and high (>29.9)), smoking status (non-smoker or current smoker) and geographic groups (described above, Fig. 1a). The significance level was set to 0.05.

## Additional Information

**How to cite this article**: Sulen, A. *et al*. Elevated monocyte phosphorylated p38 in nearby employees after a chemical explosion. *Sci. Rep.* **6**, 29060; doi: 10.1038/srep29060 (2016).

## Supplementary Material

Supplementary Information

## Figures and Tables

**Table 1:** Multiple regression analysis of monocyte p-p38 by gender, age, smoking status, geographic group and BMI (n = 301).

	n (%)	Crude MFI^B^		Adjusted		
		Mean	95% CI	Coef.	p	95% CI
Gender						
Female	128 (43)	15,297	(14,093, 16,502)	ref		
Male	173 (57)	15,207	(14,241, 16,175)	−665	0.42	(−2,283, 952)
Age (years)						
[18, 39]	112 (37)	16,213	(14,973, 17,453)	ref		
[40, 49]	79 (26)	14,668	(13,182, 16,156)	−1,456	0.14	(−3,371, 458)
[50, 67]	110 (37)	14,676	(13,436, 15,917)	−1,262	0.16	(−3,021, 496)
Smoking status						
Non-smokers	208 (69)	15,020	(14,091, 15,948)	ref		
Smokers	93 (31)	15,752	(14,468, 17,036)	667	0.43	(−982, 2,317)
Geographic^A^						
Inhabitant >20 km	104 (35)	13,878	(12,563, 15,192)	ref		
Inhabitant <6 km	105 (35)	15,505	(14,239, 16,772)	1,605	0.08	(−207, 3,418)
Employees	92 (30)	16,497	(15,183, 17,810)	2,711	0.006	(783, 4,640)
BMI						
Low [<−, 24.9]	106 (35)	14,659	(13,372, 15,945)	ref		
Medium [25, 29.9]	133 (44)	15,261	(14,194, 16,327)	897	0.31	(−828, 2,622)
High [30, −>]	62 (21)	16,218	(14,356, 18,081)	1,863	0.08	(−225, 3,952)

Means of crude values and their 95% confidence intervals (95% CI) are listed in the left part of the table, while coefficients (coef.), significance (p) and 95% confidence intervals (95% CI) from the regression analysis are listed in the right part of the table.

^A^Described in Fig. 1. Distribution of predictors in the different geographic groups is presented in Supplementary Table S1.

^B^Crude values for each subgroup of predictors are presented in boxplots in Supplementary Fig. S3.

**Table 2:** Multiple regression analysis of T-cell p-p38 by gender, age, smoking status, geographic group and BMI (n = 301).

	n (%)	Crude MFI^B^		Adjusted		
		Mean	95% CI	Coef.	p	95% CI
Gender						
Female	128 (43)	756	(715, 796)	ref		
Male	173 (57)	784	(752, 817)	7	0.81	(−47, 61)
Age (years)						
[18, 39]	112 (37)	776	(736, 816)	ref		
[40, 49]	79 (26)	796	(741, 851)	14	0.67	(−50, 78)
[50, 67]	110 (37)	751	(711, 791)	−19	0.52	(−78, 40)
Smoking status						
Non-smokers	208 (69)	749	(720, 779)	ref		
Smokers	93 (31)	824	(777, 871)	74	0.009	(19, 130)
Geographic^A^						
Inhabitant >20 km	104 (35)	779	(741, 817)	ref		
Inhabitant <6 km	105 (35)	739	(692, 786)	−34	0.27	(−95, 27)
Workers	92 (30)	803	(757, 849)	13	0.70	(−52, 77)
BMI						
Low [<−, 24.9]	106 (35)	744	(701, 789)	ref		
Medium [25, 29.9]	133 (44)	780	(742, 818)	33	0.27	(−25, 91)
High [30, −>]	62 (21)	803	(750, 856)	63	0.08	(−7, 133)

Means of crude values and their 95% confidence intervals (95% CI) are listed in the left part of the table, while coefficients (coef.), significance (p) and 95% confidence intervals (95% CI) from the regression analysis are listed in the right part of the table.

^A^Described in Fig. 1. Distribution of predictors in the different geographic groups is presented in Supplementary Table S1.

^B^Crude values for each subgroup of predictors are presented in boxplots in Supplementary Fig. S3.

**Table 3:** Gender stratified multiple regression analysis of T-cell p-p38 by age, smoking status (shown), geographic groups and BMI.

	Men				Women				Interaction
	n	β	95% CI	p	n	β	95% CI	p	p
Non-smoker	111	ref			97	ref			
Smoker	62	97	(30, 163)	0.005	31	23	(−79, 124)	0.66	0.22

Coefficients (β), 95% confidence intervals (95% CI) and significance (p) from the regression analyses are listed. Test for interactions between smoking and gender in unstratified model in far right column.

**Table 4:** Gender stratified multiple regression analysis of monocyte p-p38 by age, smoking status, geographic groups (shown) and BMI.

	Men				Women				Interaction
	n	β	95% CI	p	n	β	95% CI	p	p
>20 km	52	ref			52	ref			
<6 km	45	506	(−2,072, 3,084)	0.70	60	2,679	(36, 5,323)	0.047	0.14
Employee	76	2,683	(406, 4,960)	0.02	16	1,379	(−2,562, 5,319)	0.49	0.26

Coefficients (β), 95% confidence intervals (95% CI) and significance (p) from the regression analyses are listed. Test for interactions between geographic groups and gender in unstratified model in far right column.
