# Supplementary material for: Elevated monocyte phosphorylated p38 in nearby employees after a chemical explosion
Source: Sci Rep. 2016 Jul 6;6:29060. doi: 10.1038/srep29060 (PMC4933906; doi:10.1038/srep29060)
Supplement: Supplementary Information [file srep29060-s1.pdf]

Supplementary Information for:

Elevated monocyte phosphorylated p38 in nearby employees after a chemical explosion

Authors:

André Sulen, Stein H. L. Lygre, Sigrun M. Hjelle, Bjørg E. Hollund and Bjørn T. Gjertsen

Supplementary materials and methods

Immunophenotyping of monocyte subsets (Fig. S1)

PBMCs from blood collected in EDTA tubes from blood donors were isolated by density gradient separation (Lymphoprep™, Stemcell Technologies). Live cells were stained with Anti-CD45 FITC (clone MEM-28, EXBIO), Anti-HLA-DR Pacific Blue (clone MEM-12, EXBIO), Anti-CD16 BV605™ (clone 3G8, BioLegend) and Anti-CD14 biotin (clone M5E2, Biolegend) in combination with Strepdavidin Alexa Fluor® 647 (Molecular Probes). Acquisition and analysis was carried out as described in materials and methods in the publication.

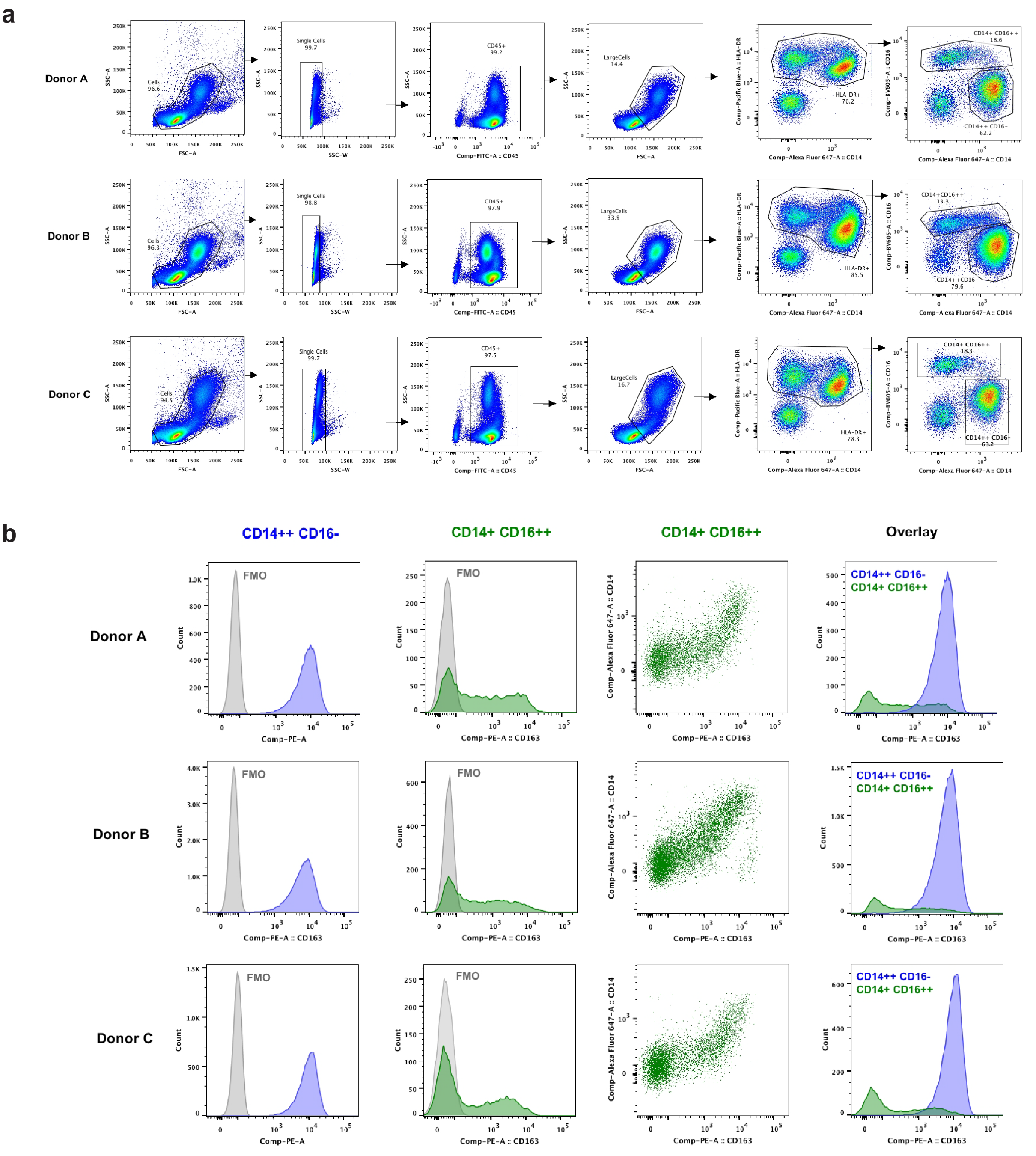

Fig. S1. CD163 is expressed predominantly in classical monocytes compared to non-classical monocytes. Live PBMCs from three healthy donors were stained with antibodies to evaluate CD163 expression in monocyte subsets. (**a**) Far right scatterplots display gating strategy of CD45+ HLA-DR+ cells into classical (CD14++ CD16-) and non-classical monocytes (CD14+ CD16++) based on CD14 and CD16 expression. (**b**) Histogram overlays and dot plots display CD163 expression in the monocyte subsets for each donor.

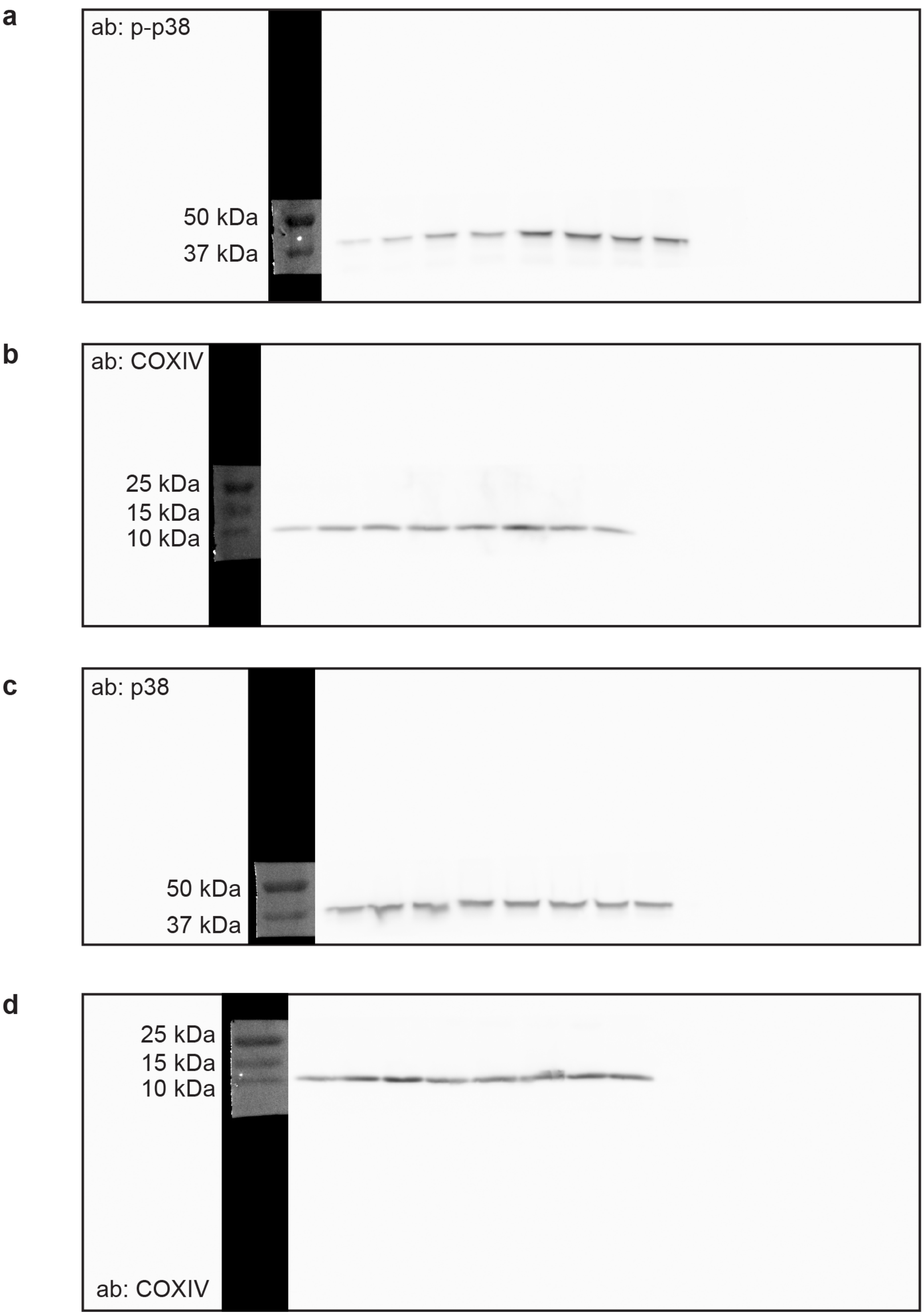

Fig. S2. Uncropped Western blots of PBMC lysates from eight individuals with high or low monocyte p-p38, probed with antibodies detecting p-p38 (**a**), total p38 (**c**) and COX IV (**b**,**d**). Reference ladder is overlaid at its correct position with band sizes indicated.

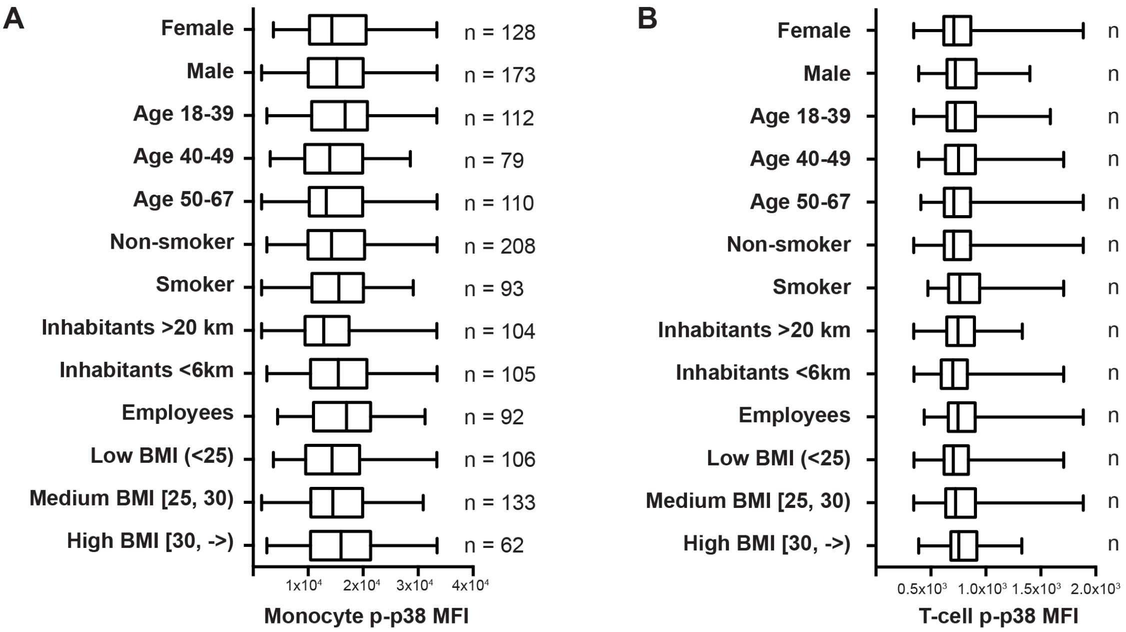

Fig. S3. Distribution of p-p38 MFIs in all predictor subgroups in regression analyses (Tables 1 & 2). Boxplots displaying p-p38 MFI values for monocytes **(a)** and T-cells **(b).** Boxes extend from 25th to 75th percentiles, whiskers display minimum to maximum values and lines in boxes represent median values.

Table S1. Demographics of the study group included in the regression analyses (Tables 1 & 2).

	**All n = 301**	**>20 km n = 104**	**<6 km n = 105**	**Employees n = 92**
Gender				
Female	128	52	60	16
Male	173	52	45	76
Age (years)				
[17, 39]	112	32	45	35
[40, 49]	79	29	22	28
[50, 67]	110	43	38	29
Smoking habits				
Non-smokers	208	76	74	58
Smokers	93	28	31	34
Geographic[1]				
Inhabitant >20km	104	104	0	0
Inhabitant <6km	105	0	105	0
Employees	92	0	0	92
BMI				
Low [<-, 24.9]	106	31	49	26
Medium [25, 29.9]	133	48	37	48
High [30, ->]	62	25	19	18

1) *cf.* Figure 1a

Table S2. Effects on mean monocyte p-p38 from gender, age, smoking status, geographic group, body mass index (BMI) and hs-CRP (n=300). Crude mean level of monocyte p-p38 and their 95% confidence intervals (95%CI) are listed in the left part of the table, while adjusted coefficients (coef.), p-values (p) and 95% confidence intervals (95%CI) are listed in the right part of the table.

	n (%)	Crude		Adjusted		
		Mean	95% CI	coef.	p	95% CI
Gender						
Female	127 (42)	15,206	(14,007, 16,407)	ref		
Male	173 (58)	15,207	(14,241, 16,175)	-607	0.46	(-2,224, 1,010)
Age (years)						
[18, 39]	112 (37)	16,213	(14,973, 17,453)	ref		
[40, 49]	79 (26)	14,668	(13,182, 16,156)	-1,415	0.15	(-3,328, 499)
[50, 67]	109 (37)	14,565	(13,333, 15,797)	-1,410	0.12	(-3,172, 352)
Smoking status						
Non-smokers	207 (69)	14,963	(14,036, 15,889)	ref		
Smokers	93 (31)	15,752	(14,468, 17,036)	661	0.43	(-985, 2,307)
Geographic[A]						
Inhabitant >20km	104 (35)	13,878	(12,563, 15,192)	ref		
Inhabitant <6km	104 (35)	15,397	(14,137, 16,657)	1,665	0.08	(-169, 3,500)
Employees	92 (30)	16,497	(15,183, 17,810)	**2,721**	**0.006**	**(796, 4,647)**
BMI						
Low [<-, 25)	106 (35)	14,659	(13,372, 15,945)	ref		
Medium [25, 30)	133 (45)	15,261	(14,194, 16,327)	821	0.35	(-910, 2,554)
High [30, ->]	61 (20)	16,045	(14,184, 17,905)	1,248	0.27	(-974, 3,470)
hsCRP[B]						
Low [0, 2]	215 (72)	14,904	(14,074, 15,733)	ref		
Medium [3, 5]	52 (17)	15,522	(13,388, 17,657)	422	0.69	(-1,644, 2,488)
High [6-21]	33 (11)	16,690	(14,041, 19,338)	1,763	0.17	(-782, 4,309)

A) Described in Fig. 1a
B) 1 individual excluded due to missing CRP data.

Table S3. Effects on mean T-cell p-p38 from gender, age, smoking status, geographic group, body mass index (BMI) and hs-CRP (n=300). Crude mean level of T-cell p-p38 and their 95% confidence intervals (95%CI) are listed in the left part of the table, while adjusted coefficients (coef.), p-values (p) and 95% confidence intervals (95%CI) are listed in the right part of the table.

	n (%)	Crude		Adjusted		
		**Mean**	**95% CI**	**coef.**	**p**	**95% CI**
Gender						
Female	127 (42)	755	(714, 796)	ref		
Male	173 (58)	784	(752, 817)	-7	0.80	(-47, 61)
Age (years)						
[18, 39]	112 (37)	776	(736, 816)	ref		
[40, 49]	79 (26)	796	(741, 851)	17	0.60	(-47, 81)
[50, 67]	109 (37)	751	(710, 791)	-22	0.47	(-81, 37)
Smoking status						
Non-smokers	207 (69)	749	(719, 779)	ref		
Smokers	93 (31)	824	(777, 871)	**73**	**0.01**	**(18, 129)**
Geographic[A]						
Inhabitant >20km	104 (35)	779	(741, 817)	ref		
Inhabitant <6km	104 (35)	738	(691, 786)	-28	0.37	(-90, 34)
Employees	92 (30)	803	(757, 849)	15	0.66	(-50, 79)
BMI						
Low [<-, 25)	106 (35)	744	(701, 789)	ref		
Medium [25, 30)	133 (45)	780	(742, 818)	29	0.32	(-29, 87)
High [30, ->]	61 (20)	803	(749, 857)	42	0.27	(-33, 117)
hsCRP[B]						
Low [0, 2]	215 (72)	757	(728, 786)	ref		
Medium [3, 5]	52 (17)	789	(721, 856)	29	0.41	(-40, 98)
High [6-21]	33 (11)	843	(763, 923)	70	0.11	(-15, 156)

A) Described in Fig. 1a
B) 1 individual excluded due to missing CRP data.

Table S4. Effects on mean monocyte p-p38 from gender, age, smoking status, geographic group, body mass index (BMI) and tear film stability (NIBUT) (n=284). Crude mean level of monocyte p-p38 and their 95% confidence intervals (95%CI) are listed in the left part of the table, while adjusted coefficients (coef.), p-values (p) and 95% confidence intervals (95%CI) are listed in the right part of the table.

	n	Crude MFI		Adjusted		
		**Mean**	**95% CI**	**coef.**	**p**	**95% CI**
Gender						
Female	120	15,074	(13,841, 16,307)	ref		
Male	164	15,262	(14,251, 16,272)	-529	0.54	(-2,224, 1,166)
Age (years)						
[18, 39]	102	15,952	(14,661, 17,242)	ref		
[40, 49]	77	14,728	(13,205, 16,251)	-1,146	0.26	(-3,142, 850)
[50, 67]	105	14,769	(13,473, 16,064)	-988	0.29	(-2,823, 847)
Smoking status						
Non-smokers	192	14,944	(13,968, 15,920)	ref		
Smokers	92	15,680	(14,390, 16,970)	700	0.42	(-988, 2,388)
Geographic[A]						
Inhabitant >20km	95	13,807	(12,414, 15,200)	ref		
Inhabitant <6km	103	15,329	(14,063, 16,596)	1,555	0.11	(-328, 3,438)
Employees	86	16,526	(15,146, 17,906)	**2,779**	**0.009**	**(712, 4,854)**
BMI						
Low [<-, 25)	100	14,397	(13,078, 15,716)	ref		
Medium [25, 30)	128	15,290	(14,191, 16,389)	1,112	0.22	(-670, 2,893)
High [30, ->]	56	16,340	(14,358, 18,323)	**2,295**	**0.04**	**(100, 4,490)**
NIBUT[B]						
Continuous (7-60s)				-8	0.73	(-53, 38)

A) Described in Fig. 1a
B) 17 individuals excluded due to missing NIBUT data.

Table S5. Effects on mean monocyte p-p38 from gender, age, smoking status, geographic group, body mass index (BMI) and airway obstruction (n=273). Airway obstruction was defined as a FEV1/FVC ratio below 0.7. Crude mean level of monocyte p-p38 and their 95% confidence intervals (95%CI) are listed in the left part of the table, while adjusted coefficients (coef.), p-values (p) and 95% confidence intervals (95%CI) are listed in the right part of the table.

	n	Crude MFI		Adjusted		
		**Mean**	**95% CI**	**coef.**	**p**	**95% CI**
Gender						
Female	112	15,222	(13,933, 16,511)	ref		
Male	161	15,163	(14,151, 16,176)	-676	0.44	(-2,390, 1,039)
Age (years)						
[18, 39]	99	16,016	(14,664, 17,368)	ref		
[40, 49]	72	14,793	(13,237, 16,349)	-940	0.36	(-2,975, 1,095)
[50, 67]	102	14,662	(13,380, 15,944)	-807	0.40	(-2,709, 1,095)
Smoking status						
Non-smokers	187	14,896	(13,918, 15,874)	ref		
Smokers	86	15,821	(14,459, 17,184)	1,020	0.26	(-747, 2,787)
Geographic[A]						
Inhabitant >20km	96	13,545	(12,182, 14,907)	ref		
Inhabitant <6km	90	15,733	(14,372, 17,095)	**2,262**	**0.03**	**(318, 4,205)**
Employees	87	16,436	(15,067, 17,805)	**3,006**	**0.003**	**(1,004, 5,008)**
BMI						
Low [<-, 25)	94	14,403	(13,014, 15,793)	ref		
Medium [25, 30)	121	15,252	(14,137, 16,368)	1,172	0.21	(-652, 2,997)
High [30, ->]	58	16,324	(14,415, 18,232)	**2,251**	**0.045**	**(52, 4,450)**
Obstruction[B]						
No	239	15,157	(14,306, 16,007)	ref		
Yes	34	15,403	(13,114, 17,692)	-211	0.87	(-2,718, 2,297)

A) Described in Fig. 1a
B) 28 individuals excluded due to missing spirometry data

Table S6. Effects on mean monocyte p-p38 from gender, age, smoking status, geographic group, body mass index (BMI) and subjective stressful life events (IES-R) (n=301). Crude mean level of monocyte p-p38 and their 95% confidence intervals (95%CI) are listed in the left part of the table, while adjusted coefficients (coef.), p-values (p) and 95% confidence intervals (95%CI) are listed in the right part of the table.

	n (%)	Crude MFI		Adjusted		
		**Mean**	**95% CI**	**coef.**	**p**	**95% CI**
Gender						
Female	128 (43)	15,297	(14,093, 16,502)	ref		
Male	173 (57)	15,207	(14,241, 16,175)	-647	0.43	(-2,270, 976)
Age (years)						
[18, 39]	112 (37)	16,213	(14,973, 17,453)	ref		
[40, 49]	79 (26)	14,668	(13,182, 16,156)	-1,475	0.13	(-3,396, 444)
[50, 67]	110 (37)	14,676	(13,436, 15,917)	-1,295	0.15	(-3,065, 474)
Smoking status						
Non-smokers	208 (69)	15,020	(14,091, 15,948)	ref		
Smokers	93 (31)	15,752	(14,468, 17,036)	666	0.43	(-986, 2,318)
Geographic[A]						
Inhabitant >20km	104 (35)	13,878	(12,563, 15,192)	ref		
Inhabitant <6km	105 (35)	15,505	(14,239, 16,772)	1,565	0.09	(-263, 3,392)
Employees	92 (30)	16,497	(15,183, 17,810)	**2,676**	**0.007**	**(736, 4,616)**
BMI						
Low [<-, 25)	106 (35)	14,659	(13,372, 15,945)	ref		
Medium [25, 30)	133 (44)	15,261	(14,194, 16,327)	887	0.31	(-842, 2,615)
High [30, ->]	62 (21)	16,218	(14,356, 18,081)	1,844	0.08	(-250, 3,938)
Total IES-R						
Low (< 24)	290 (96)	15,203	(14,436, 15,969)	ref		
High (> 23)	11 (4)	16,388	(11,835, 20,941)	768	0.71	(-3,249, 4,785)

A) Described in Fig. 1a
